# *Buccaneer* model building with neural network fragment selection

**DOI:** 10.1107/S205979832300181X

**Published:** 2023-03-28

**Authors:** Emad Alharbi, Radu Calinescu, Kevin Cowtan

**Affiliations:** a Department of Computer Science, University of York, Heslington, York YO10 5GH, United Kingdom; b Department of Chemistry, University of York, Heslington, York YO10 5DD, United Kingdom; c Department of Information Technology, University of Tabuk, Tabuk, Saudi Arabia; University of Konstanz, Germany

**Keywords:** neural networks, *Buccaneer*, model building, structure solution

## Abstract

Tracing the backbone is a critical step in protein model building, as incorrect tracing leads to poor protein models. Here, a neural network trained to identify unfavourable fragments and remove them from the model-building process in order to improve backbone tracing is presented. Moreover, a decision tree was trained to select an optimal threshold to eliminate unfavourable fragments. The neural network was tested on experimental phasing data sets from the Joint Center for Structural Genomics (JCSG), recently deposited experimental phasing data sets (from 2015 to 2021) and molecular-replacement data sets. The experimental results show that using the neural network in the *Buccaneer* protein-model-building software can produce significantly more complete protein models than those built using *Buccaneer* alone. In particular, *Buccaneer* with the neural network built protein models with a completeness that was at least 5% higher for 25% and 50% of the original and truncated resolution JCSG experimental phasing data sets, respectively, for 28% of the recently collected experimental phasing data sets and for 43% of the molecular-replacement data sets.

## Abstract 2

A neural network trained to identify unfavourable fragments and therefore improve protein model building in the *Buccaneer* software is described.

## 1. Introduction

Model-building pipelines such as *ARP*/*wARP* (Perrakis *et al.*, 1999 ▸; Langer *et al.*, 2008 ▸) and *Buccaneer* (Cowtan, 2006 ▸) start their model building by finding the backbone of the protein structure. In *Buccaneer*, this involves joining an ensemble of chain fragments in a way which maximizes the length of the resulting chain. The procedure used to find the longest chain can yield incorrect tracing because of the choice of residues that are incorrectly placed. We examined this problem by modifying the growing step output of the *Buccaneer* model-building process so that each possible tripeptide in the trace is omitted in turn from the long fragments. The protein structure built without each of these tripeptides was evaluated against the deposited structure. Identifying and removing unfavourable tripeptides improves the protein structure, because such fragments break up some paths and force the tracing algorithm to change its direction away from the correct trace.

Machine learning and neural networks play useful roles in the area of protein model building; see, for example, Bond *et al.* (2020 ▸), Alharbi *et al.* (2021 ▸) and Chojnowski *et al.* (2022 ▸). Therefore, we have developed a neural network model that identifies unfavourable tripeptides and can be used to efficiently eliminate them from protein model building before the backbone-tracing step. The neural network predicts scores indicating that these tripeptides are unfavourable based on fragment features calculated from the electron-density map and the protein model geometry. We show that backbone tracing is significantly improved by eliminating tripeptides with scores below a certain threshold as unfavourable.

## 2. Methods

### 2.1. Creating the training data sets

We used molecular-replacement (MR) data sets containing 1351 protein structures (Bond *et al.*, 2020 ▸) to create the training data sets for the neural network. These MR data sets have resolution ranges from 1.0 to 3.5 Å. For each protein structure, we ran the finding and growing steps in *Buccaneer*; the finding step to determine the likely positions of a few C^α^ atoms in the electron-density map and the growing step to grow the C^α^ atoms into longer fragments (Cowtan, 2006 ▸). The output of the growing step is a set of overlapped fragments that have different lengths. Each fragment was split into three-residue fragments, which we call tripeptides. All of these tripeptides were saved into a CIF file. The procedure for labelling each of the tripeptides as either a ‘favourable fragment’, *i.e.* those which lead to a better model in subsequent cycles, or an ‘unfavourable fragment’, which leads to a worse model in subsequent cycles, is as follows.

(i) Run *Buccaneer* for one building cycle starting from the joining step in order to build a protein structure from all the tripeptides, and compare the built structure with the deposited structure to compute the structure completeness (*i.e.* the percentage of residues matching the residues in the deposited structure with a distance of less than 1 Å and with the same residue type). This structure and its completeness provide a baseline for the later steps of our solution.
(ii) Omit one tripeptide at a time and build the protein structure as in step (i).
(iii) Compare the completeness of the protein structure obtained in step (ii) with that of the baseline structure from step (i).
(iv) If the structure from step (ii) has a higher structure completeness, label the omitted tripeptide ‘unfavourable’.
(v) Repeat steps (ii), (iii) and (iv) for the rest of the tripeptides, removing all of the tripeptides identified as ‘unfavourable’ in step (iv) from the model-building process.

As an additional step, we examine whether the tripeptides not removed by the procedure above were actually included in the protein structure. There are two reasons why a tripeptide may not be included in the structure and thus its removal would have no impact on the structure completeness.

(i) *Buccaneer* is not using tripeptides that cannot be combined into chains of at least six residues (which is the minimum length set in *Buccaneer* for tracing).
(ii) A small branch appended to a long fragment.

These tripeptides are also labelled ‘unfavourable’. Finally, all of the tripeptides that were not labelled ‘unfavourable’ after this additional step were labelled ‘favourable’.

Using the procedure above, we labelled the tripeptides in 1132 protein structures of the MR data sets, producing 822 366 favourable and 299 577 unfavourable tripeptides. A number of protein structures were not used for the following reasons, with the number of omitted protein structures reported in parentheses.

(i) Protein structures with more than 2856 tripeptides, as this is the highest number of chains that can be saved in a CIF file with a unique ID of two characters (172 protein structures).
(ii) Protein structures for which no unfavourable tripeptides were found using our procedure (22 protein structures).
(iii) Protein structures that had a very large number of tripeptides and the identification of the unfavourable tri­peptides could not be completed within 48 h, which is the maximum time that we allocated for processing each protein structure (25 protein structures).

### 2.2. Features of tripeptides

Table 1 ▸ shows the features used in training the neural network in addition to the electron-density map resolution. The following sections describe each of these features.

#### 2.2.1. Ramachandran angles

A residue is classified into either favoured or allowed regions based on the probability densities of phi (φ) and psi (ψ) (Ramachandran *et al.*, 1963 ▸). When the probability densities of φ and ψ are greater than 0.01 or 0.0005 rad^−2^, the residue is classified into favoured or allowed regions, respectively (Cowtan, 2003 ▸, 2006 ▸).

#### 2.2.2. Log-likelihood score

The log-likelihood score (LLK), also known as the density-likelihood function, is a score of possible C^α^-group positions that reflects the reproducibility of the density features of real C^α^ groups in a simulated electron-density map for a known structure and can be calculated as 

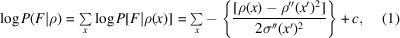

where *F* represents the electron density of the correct C^α^-group position and orientation, *x* is the coordinate in the observed density map and *x*′ is the coordinate in the search-fragment map rotated and translated to a given position and orientation in the observed map (Cowtan, 2001 ▸, 2006 ▸).

#### 2.2.3. Density score

The mean electron density for each residue in the tripeptide is calculated; the electron-density here is only calculated for the main-chain residues.

#### 2.2.4. Comparison score between tripeptides and best-matching fragments

We use the root-mean-square deviation (r.m.s.d.) between the tripeptide and best-matching fragment from the Top 500 well refined protein structure database (Lovell *et al.*, 2003 ▸). We refer to this feature as r.m.s.d. in the rest of the paper.

#### 2.2.5. Small fragment position

**Figure 1:**
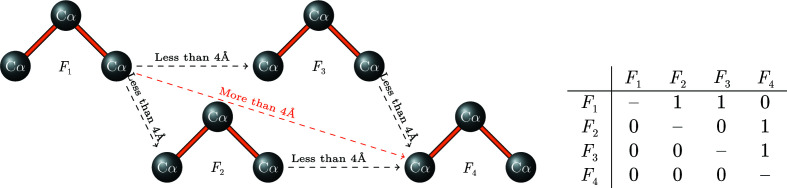
An example of four tripeptides (*F*) and the distances between them. The matrix shows when two tripeptides can be joined when the distance between them is less than 4 Å.

Another feature used by the neural network is a categorical measure that distinguishes between tripeptides located at the start, in the middle or at the end of a chain. We determine the value of this feature for a tripeptide by measuring the distance between the tripeptide and the surrounding tripeptides within a 4 Å radius of the same or even other fragments. Fig. 1 ▸ shows an example of four tripeptides and their associated joining matrix. The matrix element in row *i* and column *j* ≠ *i* of this matrix is 1 if the distance between fragments *F*_*i*_ and *F*_*j*_ is less than 4 Å and fragment *F*_*j*_ is to the right of *F*_*i*_ (meaning that *F*_*i*_ can be followed by *F*_*j*_ in a chain); otherwise, this matrix element is zero.

The tripeptides that have zeros in their corresponding columns can only be at the start of a chain and those with only zeros in their corresponding rows can only be at the end of a chain. All other tripeptides are middle fragments.

### 2.3. Neural network architecture and training

#### 2.3.1. Data-set preparation

The sets of favourable and unfavourable tripeptides from Section 2.1 were split into a training data set (containing 78.97% of the favourable fragments and 79.26% of the unfavourable fragments) and a validation data set (containing the remaining fragments). We normalized both the training data set and the validation data set by using *z*-score normalization, which is a standard practice in machine learning. This normalization ensures that the features used to train and validate the neural network have zero mean and unit standard deviation. To this end, the mean and standard deviation of every feature is calculated for the data set undergoing normalization, and the value of each data sample feature is adjusted by subtracting from it the mean and dividing the result by the standard deviation.

#### 2.3.2. Neural network architecture

The input of the neural network model is a 2849 × 14 array. The 2849 rows correspond to the largest number of tripeptides across all of the protein structures from the training and validation data sets as there are no structures with a number of tripeptides between 2849 and 2856, and the 14 columns correspond to the 14 tripeptide features that we used. The output of the neural network model is a score of the tripeptide being favourable and ranges from 0 to 1. The neural network was implemented using the Keras framework version 2.3.1 (Chollet, 2015 ▸).

Because the number of tripeptides differs between protein structures, the first layer in the neural network model is a masking layer to skip passing a data row to downstream layers when all of its values equal a mask value. This layer uses a mask value of −1 for rows from the input array for which no corresponding tripeptide is available for a protein structure, ensuring that the neural network disregards these rows.

The hidden layers contained five long short-term memory (LSTM) layers with 512 neurons in the first hidden layer and reduced in geometric sequence to 32 neurons in the last hidden layer (Hochreiter & Schmidhuber, 1997 ▸). A sigmoid function was used in the output layer, and binary cross-entropy was used for the loss function (Han & Moraga, 1995 ▸).

#### 2.3.3. Neural network training

Training of the neural network was carried out using an NVIDIA Tesla V100 32 GB SXM2 GPU server. The maximum number of epochs was set to 1000, with early stopping when the area under the curve (AUC) did not increase for ten successive epochs. The *Adam* optimizer (Kingma & Ba, 2014 ▸) was used and the learning rate was set to 0.005. To evaluate the performance of the neural network model, we used the AUC and loss function.

To evaluate the feature importance, we used permutation feature importance (Breiman, 2001 ▸), which involves shuffling the values of each feature, evaluating the neural network model obtained for the shuffled feature values and comparing it with the baseline model (the model in which the values of the features are not shuffled). As shuffling the values of the features disconnected the association with the true label, the change from the baseline model in the evaluation metrics shows the feature importance.

### 2.4. Using the neural network in *Buccaneer*

The neural network model weights and biases from Section 2.3 were extracted and saved into a CSV file, and C code was then generated for the neural network model using the Keras2c library (Conlin *et al.*, 2021 ▸). The C code was converted to C++ code for use in *Buccaneer*. As part of this work, the Keras2c library was extended to support the masking layer. The results from the Keras2c library were validated against the Keras Python framework.

**Figure 2:**
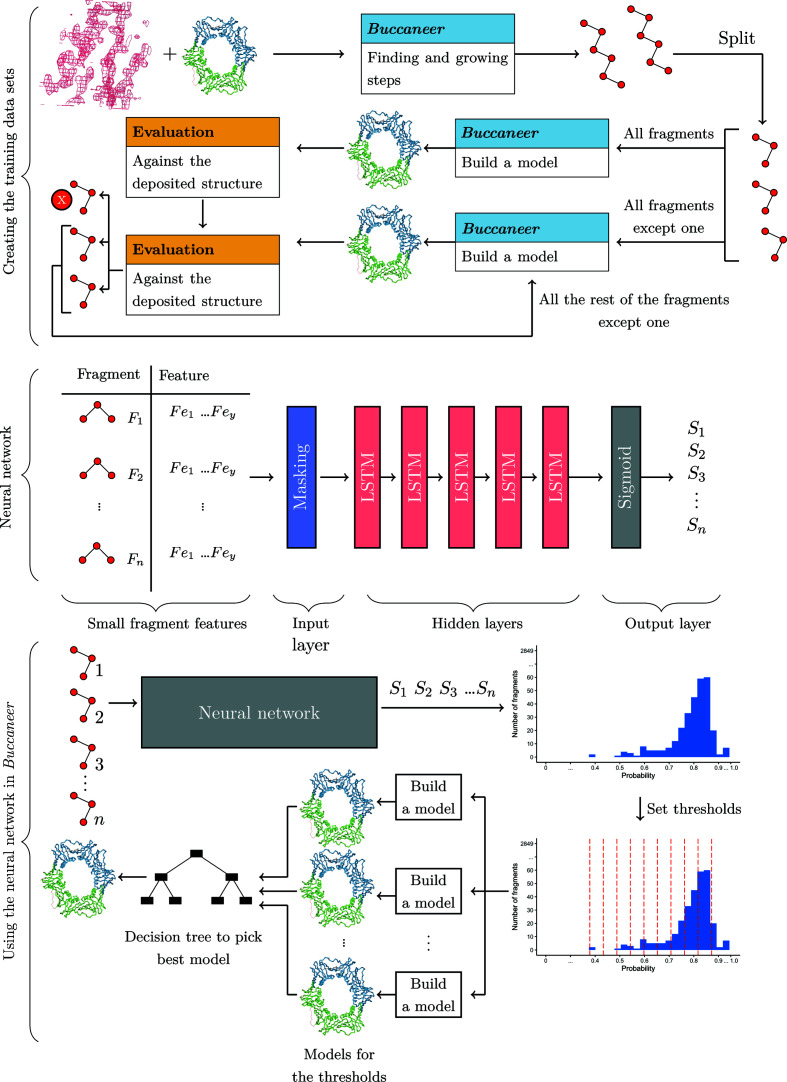
Creating the training data sets, the neural network architecture and the use of the neural network in *Buccaneer*.

As shown in Fig. 2 ▸, the neural network model is used in the joining step of *Buccaneer*, after the fragments built by *Buccaneer* in earlier steps have been split into tripeptides and before *Buccaneer* performs its tracing substep. The role of the neural network is to partition the set of tripeptides into a subset of ‘favourable’ fragments for use in the tracing substep and a subset of ‘unfavourable’ fragments that are disregarded (*i.e.* are not used for this tracing). To this end, a threshold is applied to the outputs of the neural network such that tripeptides are deemed ‘favourable’ if their associated neural network outputs (*i.e.* estimate scores of being ‘favourable’) are above this threshold. To improve the likelihood of producing a good protein model, multiple thresholds are used to generate a small set of such models and a decision tree developed by our project is employed to select the best of these models at the end of the *Buccaneer* model-building cycle.

Two mechanisms for determining the thresholds were developed. The first mechanism is to set a fixed number of thresholds (for example ten thresholds) to divide the score range into equal intervals. The second mechanism is to use the Freedman–Diaconis rule to determine the number of the thresholds based on the score distribution (Freedman & Diaconis, 1981 ▸). The Freedman–Diaconis rule can be calculated as

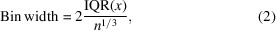

where IQR is the difference between the third and first quartiles and *n* is the number of samples. The bin width is used to split the score range and determines the thresholds.

A model will be built for each threshold by eliminating the tripeptides that have scores lower than this threshold. Moreover, we run either one or two *Buccaneer* confirmation building cycles to estimate how this protein structure will evolve in the next building cycles and then pick the best model.

A decision tree was trained to predict the best indicators to use in picking the best model (from the models built at different thresholds) using the *Weka* framework version 3.8.5 (Eibe *et al.*, 2016 ▸). Reduced error pruning (REP) was used to simplify the decision-tree size by replacing leaves with the most predicted class, and this change is kept if the performance of the tree is not negatively affected (Elomaa & Kaariainen, 2001 ▸). The decision tree is used separately from the neural network to pick the best model. The training data set for the decision tree was obtained by running *Buccaneer* using two different seeds with no neural network, as using nondefault seeds led to changes in the model. Using a nondefault seed leads to a change in the noise in the training map and causes very small changes in the LLK targets, although those changes are entirely within the uncertainties in the data. This may lead to seeds being found in very slightly different positions and orientations or, more rarely, in one seed being pushed off the bottom of the list and replaced by another.

Growing will be more affected because the small changes will sometimes be amplified as we grow a chain until a place is reached where two alternative paths are possible and the other one is selected. The outcome is that the resulting traces are similar, but usually some will differ significantly.

The difference between the *Buccaneer* evaluation indicators, *R*_work_ and *R*_free_ was calculated between models built from the same data set (Table 2 ▸). The number of residues uniquely added to a chain is determined by estimating how many chains are present (from how many independent copies of the sequence appear to have been built), allocating each sequenced chain fragment to one chain based on a score which favours compactness and completeness of each chain, and then counting how many residues of the expected sequences have actually been accounted for in this way. The *Buccaneer* evaluation indicators are interpreted in combination; for example, a model with a high number of residues uniquely allocated to a chain and a low number of residues built is better than a model with a high number of residues built and a low number of residues uniquely allocated to a chain. We deemed that the model is better when the structure completeness is at least 5% higher. The actual difference between the evaluation indicators was replaced by binary labels: ‘Y’ when the indicator is better based on Table 2 ▸ and ‘N’ otherwise. (An example of the labelling of training features is reported in the supporting information.) Under-sampling was applied to class ‘N’ to reduce it from 906 to 281 protein structures and tenfold cross-validation was used to train the decision tree. Each fold had the same proportion of each class as in the training data sets after under-sampling, as *Weka* uses stratified cross-validation by default.

The first model of these multiple models will be built from all of the fragments, as the first threshold used to partition tripeptides into ‘favourable’ and ‘unfavourable’ always has the lowest score. The number of confirmation building cycles is the remaining number of building cycles. For example, if *Buccan­eer* runs on three building cycles, we run two and one confirmation building cycles in the first and second building cycles, respectively; no confirmation building cycle is run in the third building cycle. As our neural network model is limited to 2849 tripeptides, *Buccaneer* will batch the tripeptides and run the neural network multiple times when the number of tripeptides exceeds this limit.

## 3. Results

### 3.1. Evaluation of neural network training

As is common in machine learning, we tried a wide range of neural network architectures and training hyperparameters in order to obtain a suitable neural network for our framework. For instance, we trained alternative LSTM neural networks with six layers and between 1024 and 32 neurons, and we used multiple learning rates for the training process (for example 0.001 and 0.005). Moreover, we tested a neural network of five convolutional layers and between 512 and 32 neurons and LSTM neural networks using individual fragments as input rather than an array of fragments, but the loss score was relatively high. From all of the candidate neural networks we obtained, we selected the one that had five layers (the neural network detailed in Section 2.3.2).

**Figure 3:**
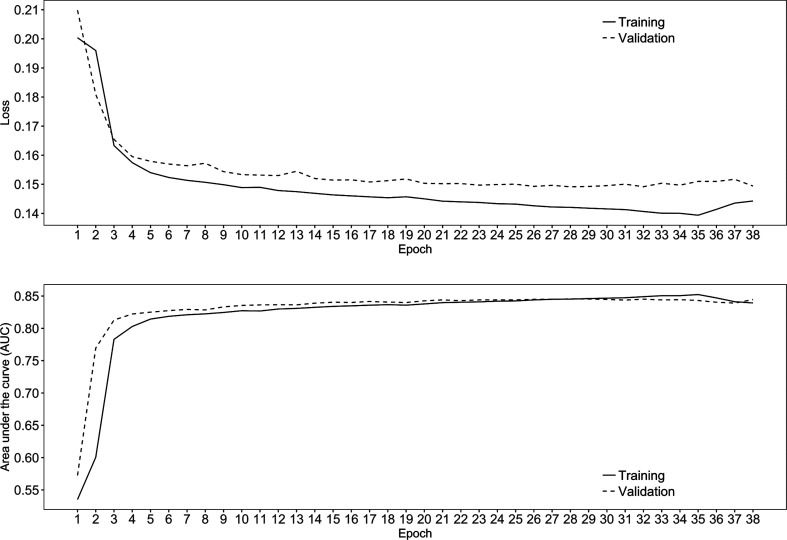
Difference in loss score and AUC between the training and validation data sets (the data sets used during model training for frequent evaluation and tuning of the model parameters) across the epochs. The best model was obtained from epoch 28 as this has the highest AUC in the validation data sets.

The training of this neural network was stopped after epoch 38, as the AUC stopped improving at epoch 28. Fig. 3 ▸ shows the AUC and loss score of the training and validation data sets across the 38 epochs. The AUC and loss score improved until epoch 28. The neural network model then started to be overfitted, as the difference in the loss score between the training and validation data sets became larger. Table 3 ▸ shows the other performance metrics, precision, recall, *F*-measure and accuracy, for the training and validation data sets at epoch 28. The neural network model from epoch 28 was used as the final neural network model.

### 3.2. Feature importance

**Figure 4:**
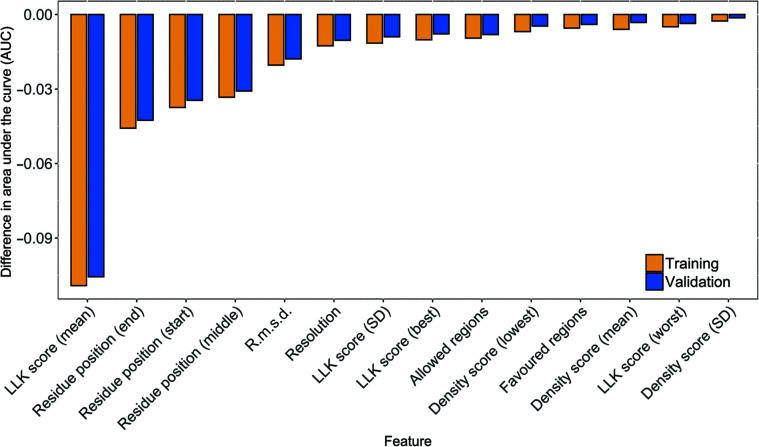
Difference between the baseline model and a model in which the feature values are shuffled to find out the importance of the features.

Fig. 4 ▸ shows the importance of features based on the change in AUC in the training and validation data sets. The application of permutation feature importance as described in Section 2.3.3 affected the AUC negatively for each of the features, decreasing it by between 0.001 and 0.11; the mean of the LLK score has the highest impact on the model. The positions of the residues decreased the performance of the model by more than 0.03 in AUC. Other features have less impact on the model performance; the SD of the density score has the lowest impact. Moreover, we trained the model without the features that have less impact on the performance and that made the loss and AUC worse, and the results are reported in the supporting information.

Overall, the features using the mean have a higher level of importance compared with the SD features for the same characteristics. For example, the mean LLK score and density score have a higher importance than the SD of the same scores. Comparing the Ramachandran angle regions showed that the model relied on the allowed regions feature rather than the favoured regions.

### 3.3. Evaluation of using the neural network in *Buccaneer*

We assessed the effect of using the neural network in *Buccaneer* for three data sets.

(i) 894 experimental phasing data sets from the Joint Center for Structural Genomics (JCSG) at the original and truncated resolutions (Bedem *et al.*, 2011 ▸; Alharbi *et al.*, 2019 ▸).
(ii) 203 newer experimental phasing data sets deposited between 2015 and 2021 and taken from the PDB.
(iii) 218 MR data sets (the remainder of the 1351 MR data sets from Section 2.1) that were not used in either training or validation of the neural network. One data set was omitted because it exceeded the 20 GB memory limit that we set for each data set.

The resolution of the JCSG experimental phasing data sets was between 1.2 and 4 Å, corresponding to 150 and 744 data sets at the original and truncated resolutions, respectively. Structure completeness, *R*_work_, *R*_free_ and structure correlation, which is the weighted *F*-map correlation between the built model and the deposited model, were considered in this evaluation; we deemed the *Buccaneer* version augmented with the neural network better when the improvement was at least 5% in the relevant measure.

We ran *Buccaneer* twice to evaluate the two methods of selecting the threshold for including tripeptides in the *Buccaneer* tracing, as described in Section 2.4. We set the maximum number of models to ten by selecting ten equidistant thresholds and only building a model for those thresholds whose use increased the number of tripeptides deemed ‘favourable’ compared with the previous threshold.

All the experiments used *Buccaneer* version 1.6.12 and the simple iterative model-building/refinement pipeline implemented in *CCP*4*i* version 7.0.045 (Winn *et al.*, 2011 ▸). We will refer to the *Buccaneer* variant that uses the neural network as ‘*Buccaneer*(NN)’ in the rest of the paper.

#### 3.3.1. Evaluation of the decision tree

The decision tree was trained using data obtained by running *Buccaneer* on JCSG experimental phasing data sets; 562 protein structures were used in training and testing after under-sampling. The trained decision tree predicted that the best model has a lower *R*_work_ and a higher number of residues uniquely allocated to a chain. The precision was 0.761, and both recall and *F*-measure were 0.760 (Table 3 ▸). Moreover, we trained the decision tree with no pruning, but the performance metrics were worse than when we used pruning.

#### 3.3.2. Experimental phasing

**Figure 5:**
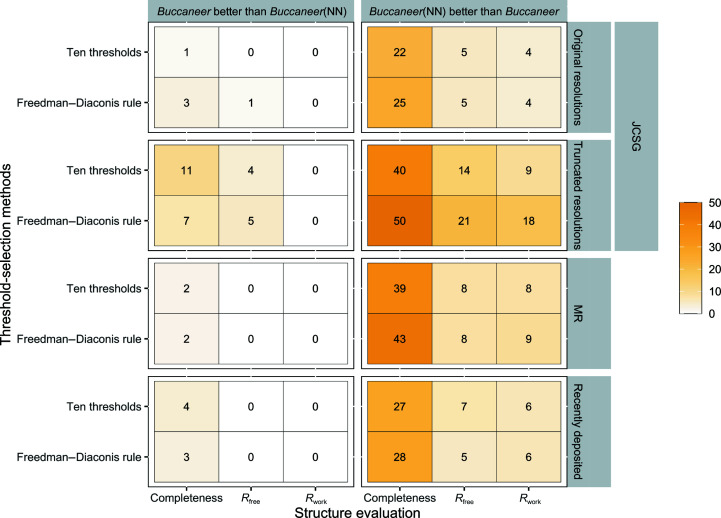
Percentage of the data sets where either *Buccaneer*(NN) or *Buccaneer* built a protein structure at least 5% better in structure completeness, *R*_work_ or *R*_free_.

Fig. 5 ▸ summarizes the results obtained for the *Buccaneer*(NN) variants with tripeptide selection based on both equidistant and Freedman–Diaconis thresholds.

This *Buccaneer*(NN) variant with equidistant thresholds built 22% and 40% of the protein structures with at least 5% higher completeness than *Buccaneer* for the original and truncated resolution JCSG data sets, respectively, compared with 1% and 11% of the data sets that were built better by *Buccaneer* without the neural network

For the original resolution, *Buccaneer*(NN) improved the *R*_work_ and *R*_free_ of 4% and 5% of the data sets, respectively, and no structure was better built by *Buccaneer*. At truncated resolutions, 9% and 14% of the protein models were built by *Buccaneer*(NN) with better *R*_work_ and *R*_work_. By comparison, only 4% of the protein structures were built with better *R*_free_ and none of the structures were built with better *R*_work_ by *Buccaneer*.

Using the Freedman–Diaconis rule to select the threshold led to *Buccaneer*(NN) building 25% and 50% of the protein models with (at least 5%) higher structure completeness and 3% and 7% with (at least 5%) lower structure completeness compared with *Buccaneer*, for the original and truncated resolution JCSG data sets, respectively. *R*_work_ and *R*_free_ improved when a fixed number of thresholds was used for the original resolution JCSG data sets, except for 1% of the data sets that were built with higher *R*_work_ and *R*_free_. However, for the truncated resolution JCSG data sets 18% and 21% of the protein structures were built with lower *R*_work_ and lower *R*_free_, respectively, and 5% of the structures were built with a higher *R*_free_ compared with *Buccaneer*; none of these structures was built with a higher *R*_work_.

For the recently deposited experimental phasing data sets, 27% and 28% of the protein models were built with higher structure completeness by *Buccaneer*(NN) using a fixed number of thresholds and the Freedman–Diaconis rule, respectively, and 4% and 3% were built with lower structure completeness by *Buccaneer*(NN) with equidistant thresholds and the Freedman–Diaconis rule, respectively, compared with *Buccaneer*. *R*_work_ improved in 6% of the data sets for both threshold-selection methods, and *R*_free_ in 7% and 5% of the data sets for the fixed number of thresholds and the Freedman–Diaconis rule, respectively; no protein structure built by *Buccaneer* had a better *R*_work_ or *R*_free_.

**Figure 6:**
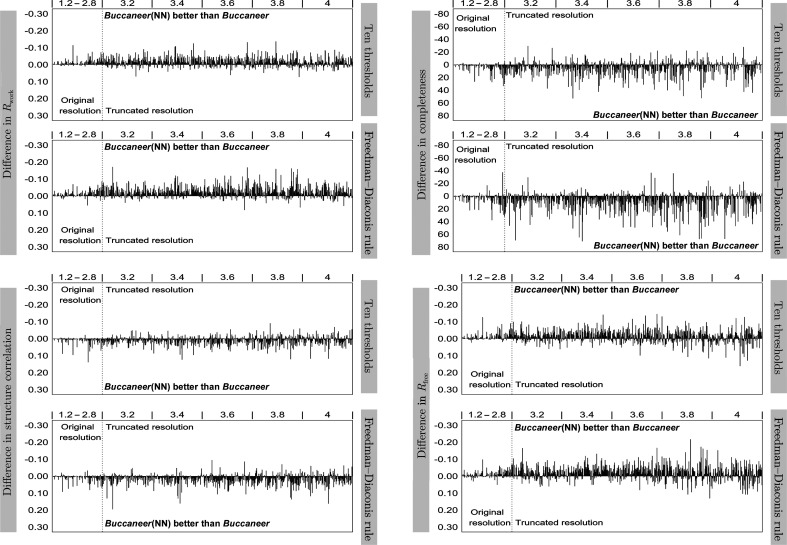
Difference in structure completeness, *R*_work_, *R*_free_ and structure correlation between *Buccaneer*(NN) and *Buccaneer*.

Figs. 6 ▸ and 7 ▸ show the results of JCSG experimental phasing for structure completeness, *R*_work_, *R*_free_ and structure correlation, and for structure completeness for the recently deposited data sets. The *R*_work_, *R*_free_ and structure correlation results for the recently deposited data sets are reported in the supporting information.

For the JCSG experimental phasing data sets, the results show multiple data sets for which the completeness significantly improved by around 50% when the neural network was used with a fixed threshold and improved even further when the Freedman–Diaconis rule was used. While *Buccaneer*(NN) did not produce better protein models for a number of data sets, it did improve the majority of the structures to different degrees. These improvements were less significant when *Buccaneer*(NN) used the Freedman–Diaconis rule.

*R*_work_ and *R*_free_ show less improvement than completeness, but *Buccaneer*(NN) did still achieve remarkable improvements in *R*_free_ and *R*_work_ for several data sets. For example, *Buccaneer*(NN) dcreased *R*_free_ for half of the data sets, with the highest improvement of around 0.10 when using the Freedman–Diaconis rule.

Structure correlation shows that the data sets built by *Buccaneer* and *Buccaneer*(NN) have similar *F*-map correlation; it is slightly better for those built by *Buccaneer*(NN) using both fixed thresholds and the Freedman–Diaconis rule. However, the data sets built by *Buccaneer*(NN) have either higher or similar structure correlation to those built by *Buccaneer* and very few have lower structure correlation. *Buccaneer*(NN) significantly improved some of the data sets; for example, the structure correlation of one data set increased by around 0.20 when using the Freedman–Diaconis rule.

For the recently deposited data sets, *Buccaneer*(NN) only produced slight improvements for the protein structures that had already been built by *Buccaneer* at resolutions between 1.0 and 2.0 Å. However, the structures built from data sets worse than 2 Å by *Buccaneer* were improved when built by *Buccaneer*(NN). Only a few protein structures were built with slightly lower completeness by *Buccaneer*(NN) compared with *Buccaneer*.

**Figure 8:**
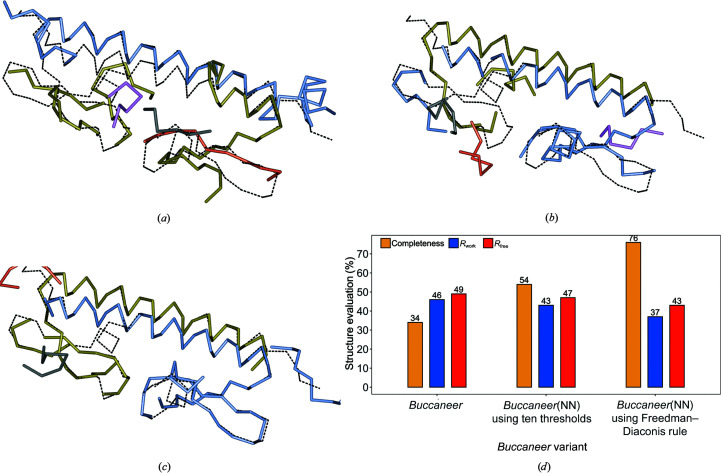
A protein structure built by *Buccaneer* and *Buccaneer*(NN) compared with the deposited structure, with the chains of the deposited structure depicted as dashed bonds. (*a*) The structure built by *Buccaneer*. (*b*, *c*) The protein structure built by *Buccaneer*(NN) using ten thresholds and the Freedman–Diaconis rule, respectively. (*d*) The structure completeness, *R*_work_ and *R*_free_ achieved by *Buccaneer* and the two *Buccaneer*(NN) variants. The PDB code for the structure is 6hcz and its resolution is 2.3 Å.

**Figure 9:**
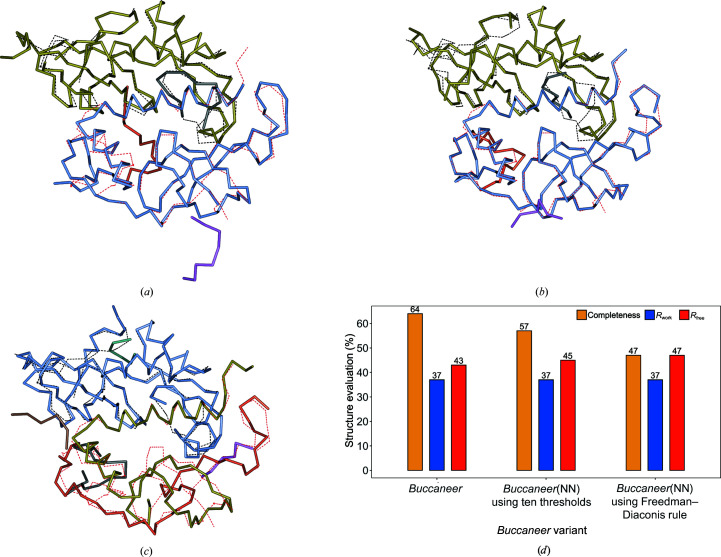
A protein structure built by *Buccaneer* and *Buccaneer*(NN) compared with the deposited structure. The chains of the deposited structure are depicted as dashed bonds. (*a*) The structure built by *Buccanee*r. (*b*, *c*) The protein structure built by *Buccaneer*(NN) using ten thresholds and the Freedman–Diaconis rule, respectively. (*d*) The structure completeness, *R*_work_ and *R*_free_ of the *Buccaneer* variant. The PDB code for the structure is 2gnr and its truncated resolution is 3.2 Å.

We illustrate the use of *Buccaneer*(NN) in Figs. 8 ▸ and 9 ▸, which depict two protein structures built by *Buccaneer* and by our two *Buccaneer*(NN) variants. To provide an impartial view, we present both a protein structure whose modelling is improved by *Buccaneer*(NN) (PDB entry 6hcz; Fig. 8 ▸) and a protein structure that *Buccaneer* builds with better results (PDB entry 2gnr; Fig. 9 ▸). Thus, for PDB entry 6hcz *Buccaneer*(NN) using the Freedman–Diaconis rule increased the structure completeness by 42%, while for PDB entry 2gnr the structure completeness decreased by 17% when *Buccaneer*(NN) was used.

#### 3.3.3. MR

As shown in Fig. 5 ▸, *Buccaneer*(NN) with a fixed number of thresholds produced protein models with (at least 5%) better completeness, *R*_work_ and *R*_free_ than *Buccaneer* for 39%, 8% and 8% of the data sets, respectively. By comparison, *Buccaneer* built protein structures with better completeness for only 2% of the data sets, respectively; no protein structure built by *Buccaneer* had a better *R*_work_ or *R*_free_ than the corresponding structure built by *Buccaneer*(NN). Using the Freedman–Diaconis rule to select the threshold, 43%, 9% and 8% of the MR data sets were built with better completeness, *R*_work_ and *R*_free_, respectively, by *Buccaneer*(NN), compared with only 2% of the MR data sets that were built with higher structure completeness by *Buccaneer*; no structure was built with (at least 5%) worse *R*_work_ or *R*_free_ by *Buccaneer*(NN).

**Figure 10:**
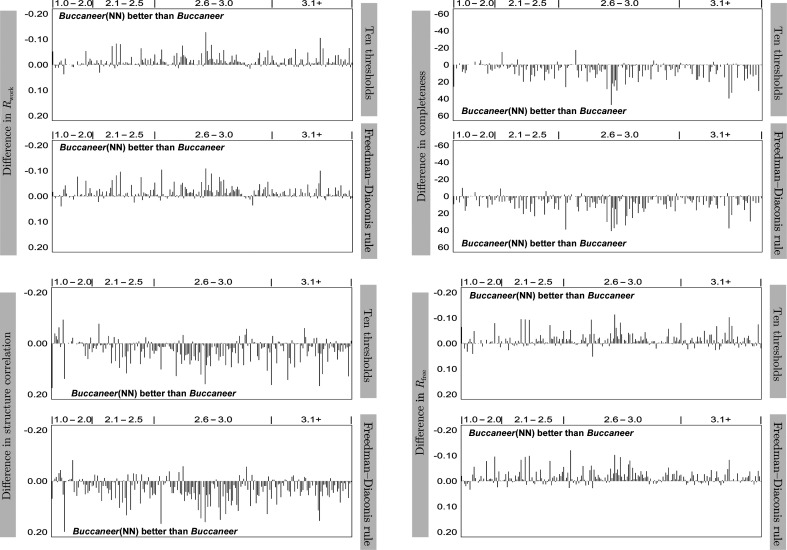
Difference in structure completeness, *R*_work_, *R*_free_ and structure correlation between the *Buccaneer*(NN) and *Buccaneer* variants using ten thresholds and the Freedman–Diaconis rule for the MR data sets. The regions where *Buccaneer*(NN) is better than *Buccaneer* (either below or above the zero point) are indicated in the diagrams.

Fig. 10 ▸ shows the same analysis for the MR data sets as in Fig. 6 ▸. The results obtained for the individual data sets show multiple significant improvements that are achieved by *Buccaneer*(NN); for example, *Buccaneer*(NN) with a fixed number of thresholds improved the completeness of one protein structure by around 40% and decreased the *R*_free_ of another protein structure by around 0.10. Moreover, *Buccaneer*(NN) improved the structure correlation for the majority of the data sets. However, very few are built with lower structure correlation compared with *Buccaneer*.

### 3.4. Evaluation of execution times

**Figure 11:**
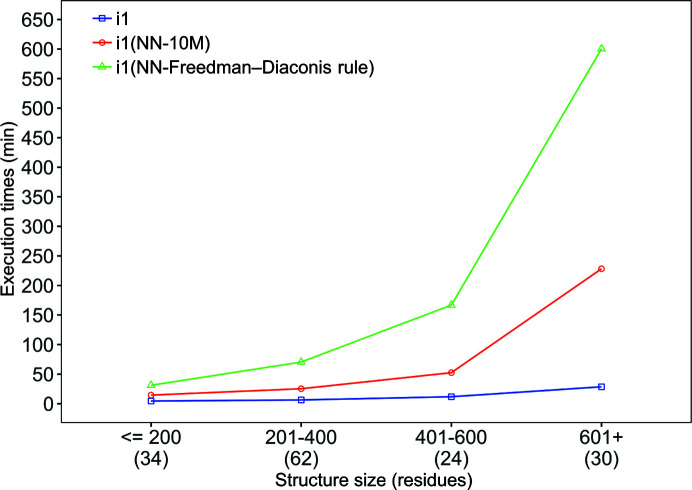
Mean execution time of *Buccaneer* and *Buccaneer*(NN) for the original JCSG data sets. The structure sizes are grouped into classes and the number of data sets in each class is reported below the graph.

Fig. 11 ▸ shows the mean *Buccaneer* and *Buccaneer*(NN) execution times for the original JCSG data sets. We ran both *Buccaneer* variants using a 173-node high-performance cluster with 7024 Intel Xeon Gold/Platinum cores and a total memory of 42 TB. For small structures, *Buccaneer*(NN) is three and seven times slower than *Buccaneer* when using fixed thresholds and the Freedman–Diaconis rule, respectively. For example, a small structure was built by *Buccaneer* within around 6 min; in comparison, *Buccaneer*(NN) using fixed thresholds and the Freedman–Diaconis rule took around 20 and 39 min, respectively. However, this execution time increased to become eight times slower than *Buccaneer* when large structures were built using the *Buccaneer*(NN) variant with fixed thresholds and 21 times slower when using the Freedman–Diaconis rule.

## 4. Discussion

A new method to improve the backbone-tracing step of protein structure-building software by using a neural network has been presented. As no training data sets were available, we created training data sets and used them in neural network training and validation. Moreover, two experimental phasing data sets were used in evaluation.

Comparing the loss score of the different neural network architectures shows that LSTM layers are more capable of learning the dependency between the tripeptides compared with convolutional layers. Moreover, a reduction in the number of LSTM layers from six to five improved the learning by the neural network, which may suggest that a larger neural network needs more data for learning.

The evaluation of the feature importance in determining favourable and unfavourable tripeptides yielded unexpected results. In particular, the r.m.s.d. of the data sets has lower importance than the residue position type. In contrast, the LLK score (used in *Buccaneer* to decide when fragments stop growing) has the greatest importance in discriminating between favourable and unfavourable tripeptides among all of the other features in protein structure building. A comparison of the impact of the mean and the SD of the features shows that the mean of a feature has higher importance than its SD.

Optimizing the threshold used to select the tripeptides used in the tracing step of protein structure building is key to achieving good neural network performance. The imbalance of the feature data makes this particularly challenging. In this paper, we addressed the threshold-tuning problem by trying several thresholds obtained by using both a fixed number of equidistant thresholds and the Freedman–Diaconis rule. Evaluation of the two threshold methods shows that the Freedman–Diaconis rule is more effective for data sets with lower resolutions; for example, the truncated resolution data sets, which all had resolutions worse than 3.1 Å, improved in their structure completeness more than the original resolution data sets.

Training a decision tree to predict the best indicators for selecting the best protein structure from a set of protein structures showed that *R*_work_ and the residues that are uniquely allocated to a chain perform best in reflecting the improvement in the structure completeness. Running *Buccaneer* on different seeds to the default seeds led to changes in the fragments and therefore to changes in the structure completeness. However, 203 newer experimental phasing data sets were used in the evaluation in order to eliminate the potential bias due to using the JCSG experimental phasing data sets in the training of the decision tree.

We use confirmation cycles (running a few cycles ahead to evaluate protein structure quality) because we are not necessarily interested in the correctness of the intermediate protein structure, but rather in the quality of the final protein structure arising from this intermediate protein structure. For our purposes, the best intermediate protein structure is that which will later lead to the most plausibly complete protein structure.

The systematic evaluation shows that completeness, *R*_work_, *R*_free_ and structure correlation are significantly improved by *Buccaneer*(NN). Moreover, our neural network has shown significant improvements even when tested on MR protein structures from *PDB-REDO*, which are likely to have fewer building mistakes than PDB structures (the results of using *PDB-REDO* protein structures are reported in the supporting information).

For MR data sets, we noticed that *Buccaneer*(NN) significantly improved the structures that *Buccaneer* built from data sets at resolutions between 1.0 and 3.0 Å. This may suggest that structures with resolutions worse than 3.0 Å have no or few favourable fragments and therefore the use of the neural network cannot improve them. The problem needs to be addressed by extending the neural network to build favourable fragments itself instead of only using those built by *Buccaneer*.

*Buccaneer*(NN) achieved higher levels of improvement in structure completeness than in *R*_work_, *R*_free_ and structure correlation. This may be due to our use of structure complete­ness as an improvement measure when the training data sets for the neural network were created. In future work, this will be addressed by creating training data sets based on the structure completeness, *R*_work_, *R*_free_ and structure correlation, and training a new version of the neural network also using training methods that take class imbalance into account.

## 5. Data and methods

The software is now available as both source code and in executable form as a part of the 8.0.009 update to the *CCP*4 software suite. Pipeline developers are evaluating how to best incorporate the software into user-facing automated model building pipelines. 

## Supplementary Material

Supporting information including Supplementary Figures and Table. DOI: 10.1107/S205979832300181X/di5061sup1.pdf

## Figures and Tables

**Figure 7:**
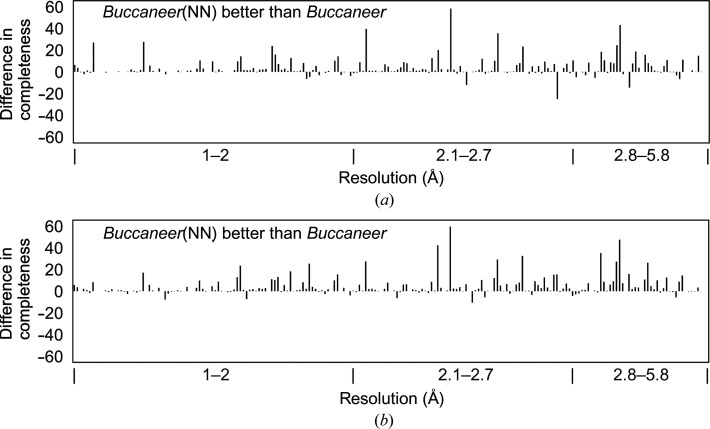
Difference in structure completeness between *Buccaneer* and the *Buccaneer*(NN) variants for the recently deposited experimental phasing data sets. (*a*) *Buccaneer*(NN) using ten thresholds. (*b*) *Buccaneer*(NN) using the Freedman–Diaconis rule. The regions in which *Buccaneer*(NN) is better than *Buccaneer* are indicated in the diagrams.

**Table 1:** Features used in training the neural network in addition to the electron-density map resolution Mean, SD, highest and lowest were calculated for the features when applicable and each was used as a separate feature.

Feature	Mean	SD	Highest	Lowest	Categorical values (0 or 1)	Single value
Ramachandran angles in favoured regions					Yes	
Ramachandran angles in allowed regions					Yes	
Log-likelihood score (LLK)	Yes	Yes	Yes	Yes		
Density score	Yes	Yes		Yes		
Root-mean-square deviation (r.m.s.d.)						Yes
Is a tripeptide at the start of a chain?					Yes	
Is a tripeptide in the middle of a chain?					Yes	
Is a tripeptide at the end of a chain?					Yes	

**Table 2:** Protein structure evaluation indicators: *Buccaneer* indicators, *R*_work_ and *R*_free_ Whether a higher or lower value of the indicator is better is indicated.

Indicator	Optimal
Longest fragment	Higher
No. of residues built	Lower
No. of fragments	Lower
No. of sequenced residues	Higher
No. of residues uniquely allocated to a chain	Higher
Completeness by residues	Higher
Completeness by chain	Higher
*R*_work_	Lower
*R*_free_	Lower

**Table 3:** Data split and the performance metrics precision, recall, *F*-measure, accuracy, loss and area under the curve (AUC) for the neural networks and the decision tree

Predictive model	Data sets	Data split	Precision	Recall	*F*-measure	Accuracy	Loss	AUC
LSTM neural networks	Training	66%	0.8315	0.9225	0.8746	0.8064	0.1421	0.8453
	Validation	33%	0.8390	0.9137	0.8748	0.8075	0.1491	0.8455
Decision tree	Cross-validation	Tenfold	0.761	0.760	0.760	0.7597	—	—
