# Supplementary material for: Buccaneer model building with neural network fragment selection
Source: Acta Crystallogr D Struct Biol. 2023 Mar 28;79(Pt 4):326–38. doi: 10.1107/S205979832300181X (PMC10071564; doi:10.1107/S205979832300181X)
Supplement: Supplementary file 1 [file d-79-00326-sup1.pdf]

Acta Cryst D STRUCTURAL BIOLOGY

Volume 79 (2023)

Supporting information for article:

Buccaneer model building with neural network fragment selection

Emad Alharbi, Radu Calinescu and Kevin Cowtan

S1. The decision tree training

S1.1 The training data sets

We run Buccaneer using two different s eeds, 1 0 a nd 2 0, w ith t he d efault parameters as set by the Buccaneer developers. The experiment ran on a 173-node high-performance cluster with 7024 Intel Xeon Gold/Platinum cores and a total memory of 42 TB. Buccaneer built 1187 protein structures using each seed. The Buccaneer's indicators and R-work and R-free were obtained for each model for the two seeds. We compared each model evaluation indicator and replaced the actual value with the label 'N' or 'Y' when the model built using seed 10 was better. We deemed the protein model built using seed ten is better when the model's completeness is at least %5 higher than when the model built using seed 20 and labelled either 'N' or 'Y' (Figure S1).

PDB	Longest fragment	Residues built	...	Completeness
1O6A	88	172	...	98.21
1VJZ	88	324	...	91.38
1VK2	157	197	...	95.79
⋮	⋮	⋮	⋮	⋮

(a) Protein models built using seed 10

PDB	Longest fragment	Residues built	...	Completeness
1O6A	89	173	...	99.40
1VJZ	153	320	...	84.31
1VK2	191	191	...	97.37
⋮	⋮	⋮	⋮	⋮

(b) Protein models built using seed 20

Labelled training features

PDB	Longest fragment	Residues built	...	Completeness
1O6A	N	N	...	N
1VJZ	N	Y	...	Y
1VK2	N	Y	...	Y
⋮	⋮	⋮	⋮	⋮

(c) The labelled training features and the predicted label

Fig. S1. The protein models evaluation indicators were built using the two seeds. (a) The protein models were built using the seed 10. (b) The protein models were built using seed 20. (c) The labelled training features and the predicted label where each evaluation indicator is replaced by either 'N' or 'Y' based on the difference between the same evaluation indicator when the model was built using seeds 10 and 20 with a difference is that the improvement should be at least 5% to be labelled 'Y'.

S2. The performance of the neural networks with and without the features that contributed less than 0.01 in the model performance.

Table S1. *The performance metrics; precision, recall, F-measure, accuracy, loss and Area Under the Curve (AUC) for the neural networks with and without the features that contributed less than 0.01 in the model performance.*

**Predictive model**	**Data sets**	**Precision**	**Recall**	**F-Measure**	**Accuracy**	**Loss**	**AUC**
LSTM neural networks (all features)	Training	0.8315	0.9225	0.8746	0.8064	0.1421	0.8453
	Validation	0.8390	0.9137	0.8748	0.8075	0.1491	0.8455
LSTM neural networks (with dropping some features)	Training	0.8274	0.9248	0.8734	0.8037	0.1448	0.8374
	Validation	0.8241	0.9362	0.8766	0.8061	0.1514	0.8401

S3. Comparison of structure completeness, R-work, R-free and structure correlation between Buccaneer and Buccaneer with neural network for the JCSG experimental phasing data sets

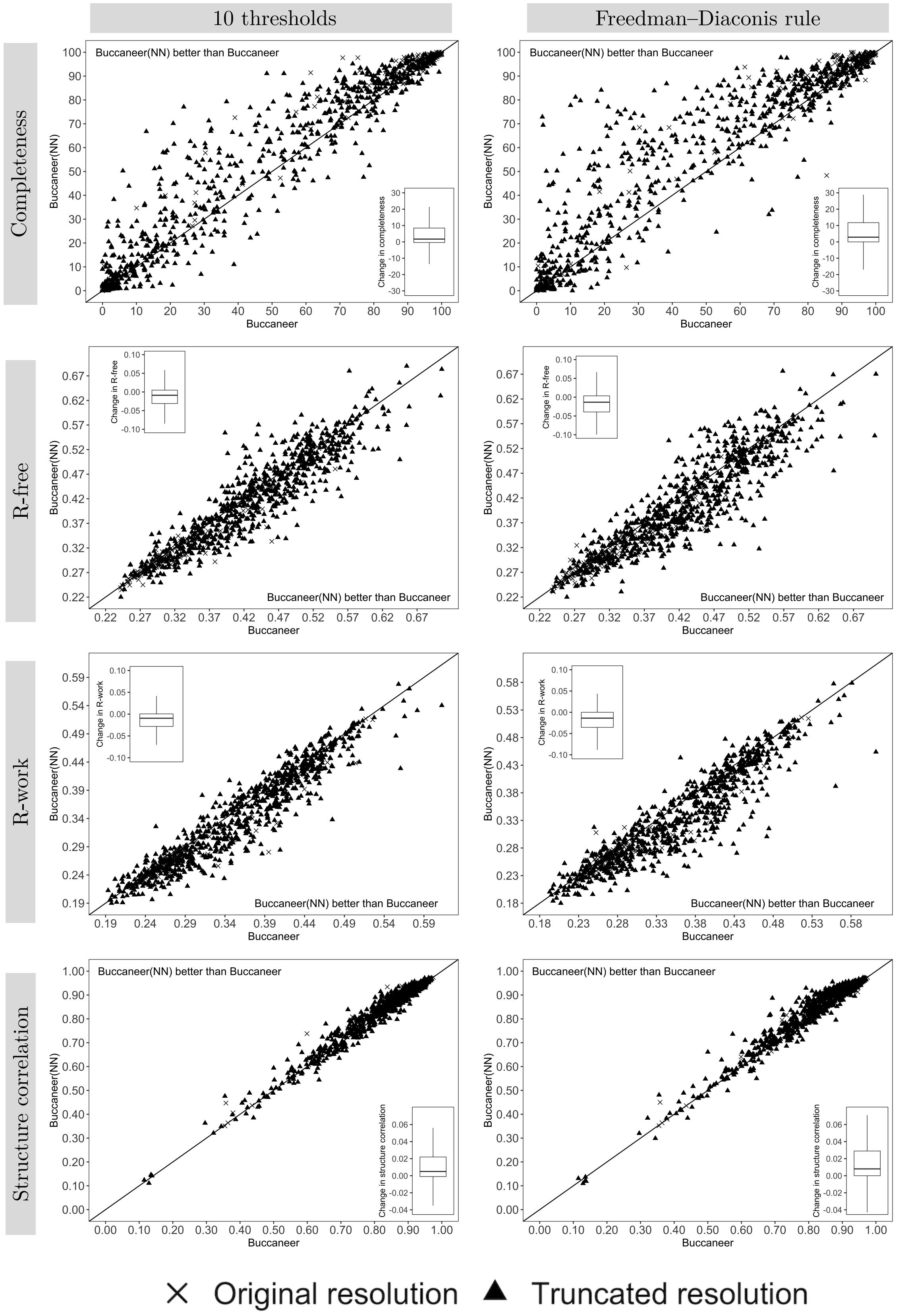

Fig. S2. Comparison of structure completeness, R-work, R-free and structure correlation between Buccaneer and the Buccaneer with neural network (Buccaneer(NN)) variants using ten thresholds and the Freedman–Diaconis rule, for the JCSG experimental phasing data sets with original and truncated resolutions. The regions where Buccaneer(NN) is better than Buccaneer (either below or above the diagonal) are indicated in the diagrams. The inset boxplot depicts the difference in the four evaluation indicators achieved by Buccaneer(NN) and Buccaneer.

S4. Comparison of structure completeness, R-work, R-free and structure correlation between Buccaneer and Buccaneer with neural network for the recently deposited experimental phasing data sets

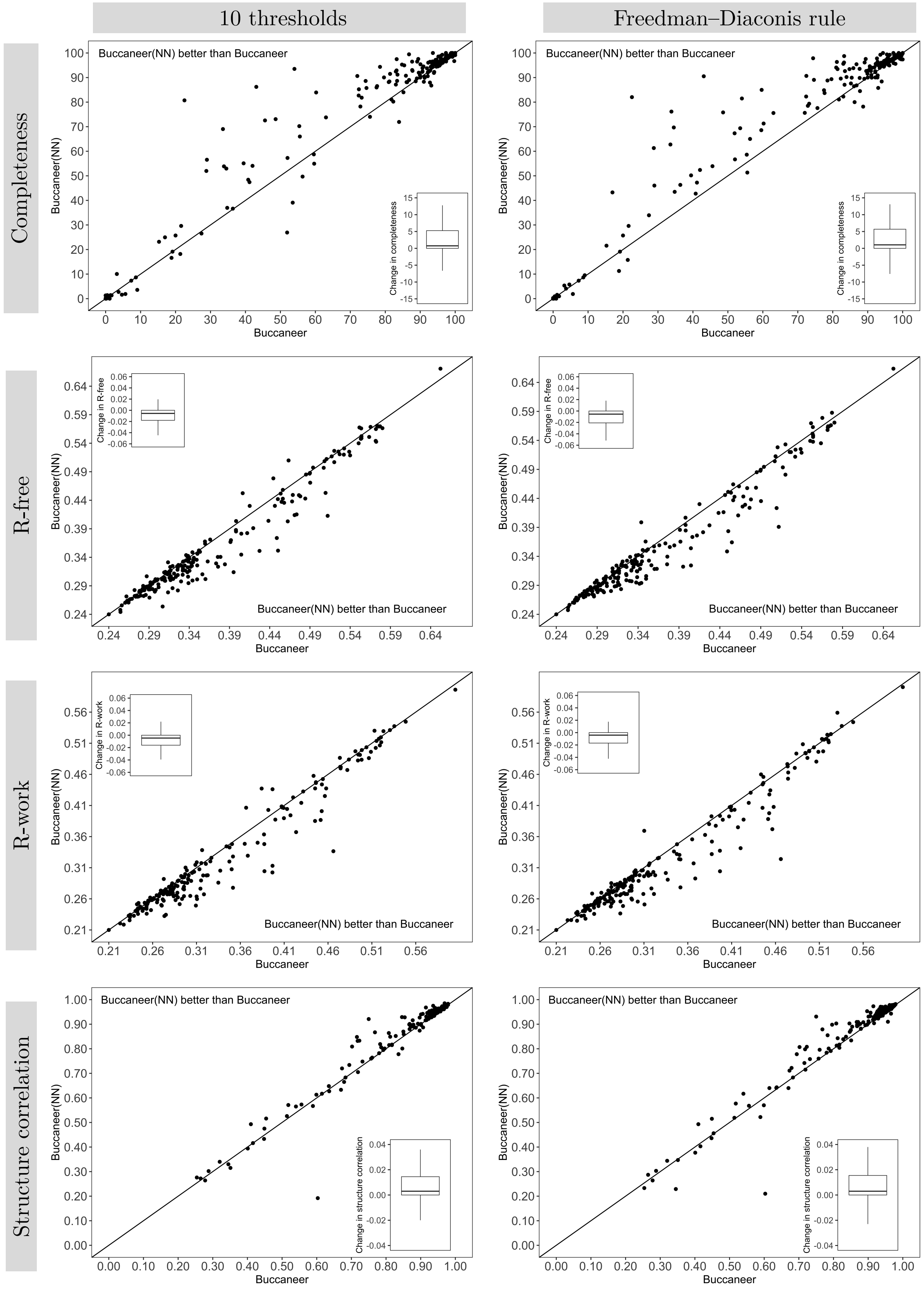

Fig. S3. Comparison of structure completeness, R-work, R-free and structure correlation between Buccaneer and Buccaneer with neural network (Buccaneer(NN)) using ten thresholds and Freedman–Diaconis rule for the recently deposited experimental phasing data sets. The results where Buccaneer(NN) is better than Buccaneer either below or above the diagonal is indicated in the figures. The inset boxplot depicts the difference in the four evaluation indicators achieved by Buccaneer(NN) and Buccaneer.

S5. Comparison of structure completeness, R-work, R-free and structure correlation between Buccaneer and Buccaneer with neural network for the MR data sets

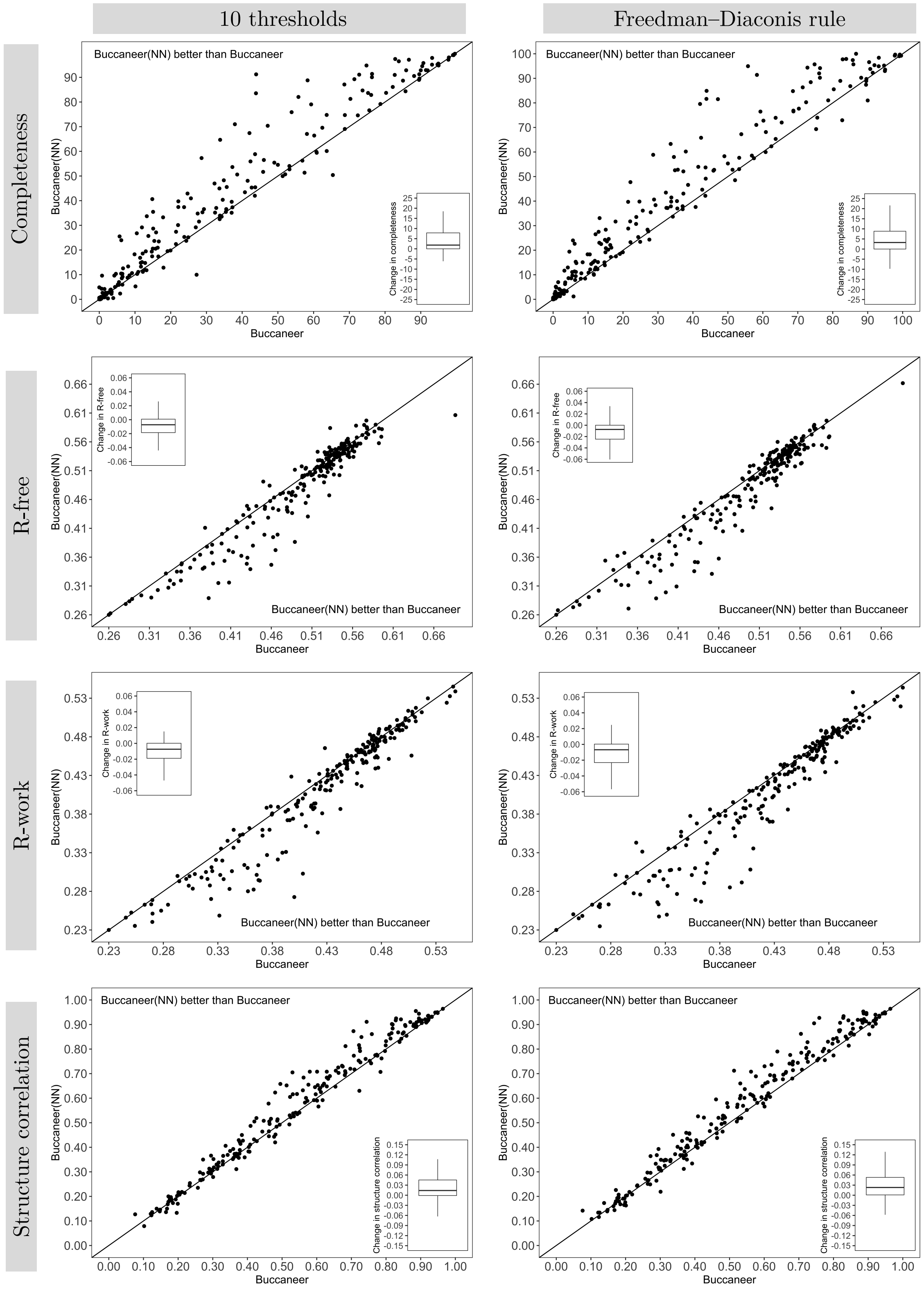

Fig. S4. Comparison of structure completeness, R-work, R-free and structure correlation between Buccaneer and Buccaneer with neural network (Buccaneer(NN)) using ten thresholds and Freedman–Diaconis rule for the MR data sets. The results where Buccaneer(NN) is better than Buccaneer either below or above the diagonal is indicated in the figures. The inset boxplot depicts the difference in the four evaluation indicators achieved by Buccaneer(NN) and Buccaneer.

S6. Comparison of structure completeness, R-work and R-free between Buccaneer and Buccaneer with neural network for the MR data sets using MR model from PDB and PDB-REDO

S6.1 Data sets

The MR models were downloaded from PDB-REDO same as in PDB data sets. PDBSET was used to extract the target chain from the PDB-REDO model. GESAMT was used with the PDB model as a reference and PDB-REDO as the moving model rather than repeating the molecular replacement. REFMAC was run for ten cycles to refine the PDB-REDO models. This provides a clear comparison of the impact of using the PDB-REDO structures on model building by eliminating any differences due to changes in the MR results.

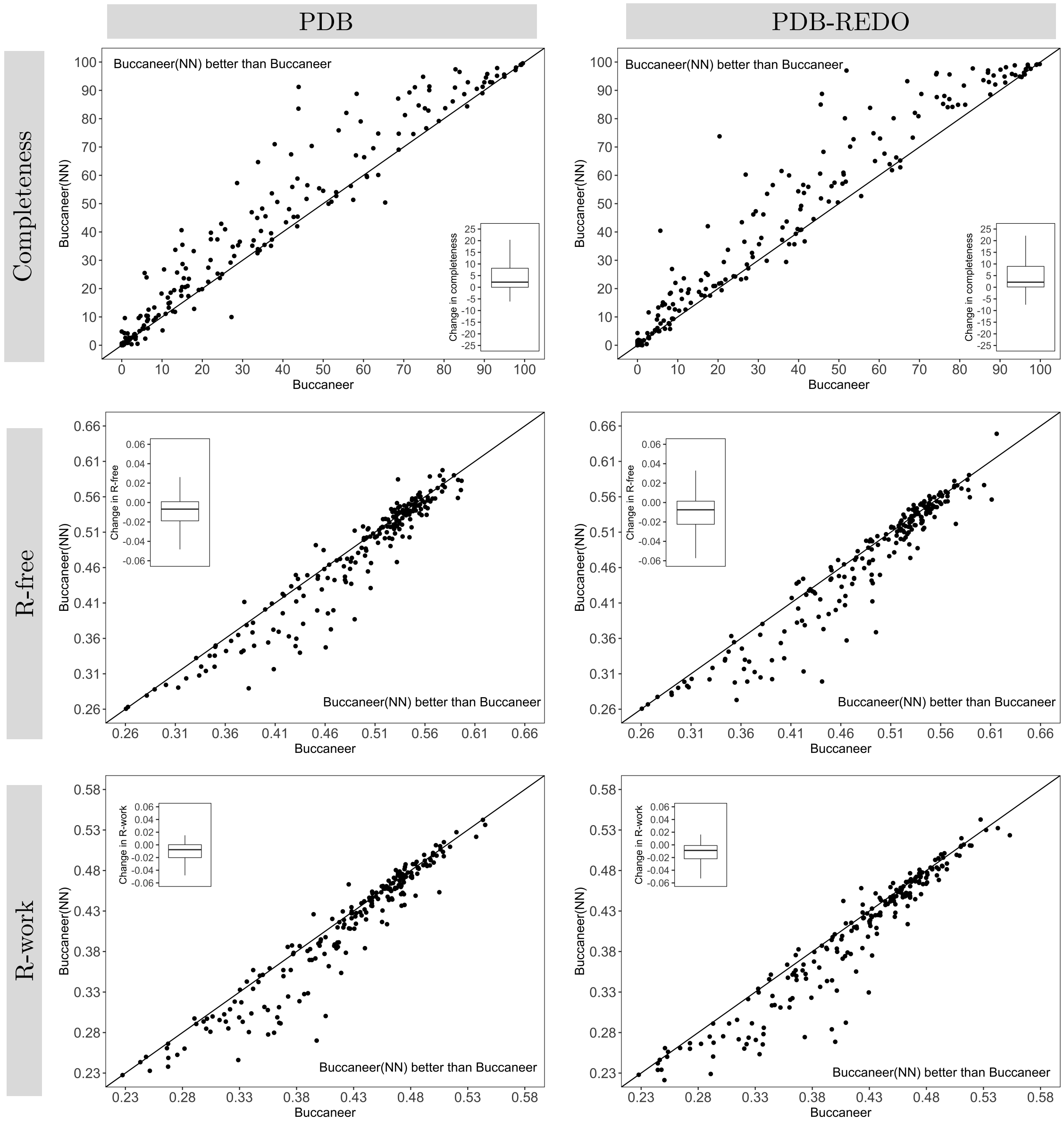

Fig. S5. Comparison of structure completeness, R-work and R-free between Buccaneer and Buccaneer with neural network (Buccaneer(NN)) using ten thresholds and MR model from PDB and PDB-REDO model. The results where Buccaneer(NN) is better than Buccaneer either below or above the diagonal is indicated in the figures. The inset boxplot depicts the difference in the four evaluation indicators achieved by Buccaneer(NN) and Buccaneer.
